# How the speed of word finding depends on ventral tract integrity in primary progressive aphasia

**DOI:** 10.1016/j.nicl.2020.102450

**Published:** 2020-09-29

**Authors:** Nikki Janssen, Ardi Roelofs, Margot Mangnus, Joanna Sierpowska, Roy P.C. Kessels, Vitória Piai

**Affiliations:** a Radboud University, Donders Institute for Brain, Cognition and Behaviour, Nijmegen, the Netherlands; b Radboud University Medical Center, Donders Centre for Medical Neuroscience, Department of Medical Psychology, Nijmegen, the Netherlands; c Vincent van Gogh Institute for Psychiatry, Venray, the Netherlands

**Keywords:** Primary progressive aphasia, Word finding, Picture-word interference, Diffusion MRI, White matter

## Abstract

Primary progressive aphasia (PPA) is a clinical neurodegenerative syndrome with word finding problems as a core clinical symptom. Many aspects of word finding have been clarified in psycholinguistics using picture naming and a picture-word interference (PWI) paradigm, which emulates naming under contextual noise. However, little is known about how word finding depends on white-matter tract integrity, in particular, the atrophy of tracts located ventrally to the Sylvian fissure. To elucidate this question, we examined word finding in individuals with PPA and healthy controls employing PWI, tractography, and computer simulations using the WEAVER++ model of word finding. Twenty-three individuals with PPA and twenty healthy controls named pictures in two noise conditions. Mixed-effects modelling was performed on naming accuracy and reaction time (RT) and fixel-based tractography analyses were conducted to assess the relation between ventral white-matter integrity and naming performance. Naming RTs were longer for individuals with PPA compared to controls and, critically, individuals with PPA showed a larger noise effect compared to controls. Moreover, this difference in noise effect was differentially related to tract integrity. Whereas the noise effect did not depend much on tract integrity in controls, a lower tract integrity was related to a smaller noise effect in individuals with PPA. Computer simulations supported an explanation of this paradoxical finding in terms of reduced propagation of noise when tract integrity is low. By using multimodal analyses, our study indicates the significance of the ventral pathway for naming and the importance of RT measurement in the clinical assessment of PPA.

## Highlights

• Noise words influence naming time, but not accuracy, more in PPA than in controls.
• Noise effect difference between PPA and controls reflects ventral tract integrity.
• The noise effect is smaller when ventral tract integrity is lower in the individuals with PPA.
• Simulations reveal that propagation of noise is reduced when tract integrity is low.

## 1 Introduction

Primary progressive aphasias (PPA) is a clinical syndrome characterised by progressive decline in language abilities caused by neurodegeneration of the language network in the brain (Mesulam, 1982). PPA is often subdivided into three different variants based on specific cognitive and neuroimaging features (Gorno-Tempini et al., 2011): semantic (sv-PPA), nonfluent/agrammatic (nfv-PPA), and logopenic (lv-PPA). Although these variants present with different types of language impairment, word-finding disturbance is one of the earliest and most prominent symptoms in all PPA variants, clinically assessed by examining picture naming difficulty. In addition to the word finding deficit, sv-PPA is characterised by word comprehension problems and loss of semantic knowledge, due to anterior temporal atrophy. Nfv-PPA is characterised by motor speech problems and agrammatism, related to atrophy of the left inferior frontal gyrus. Lv-PPA is characterised by phonological errors in naming and spontaneous speech, and impaired repetition of phrases and sentences, linked to left posterior temporal and inferior parietal atrophy (Gorno-Tempini et al., 2011, Grossman, 2010, Grossman, 2018).

Most studies investigating word finding difficulties in PPA have used traditional neuropsychological tests that focus on the accuracy of picture naming. Such tests, however, do not enable a more fine-grained analysis of naming as a complex multi-stage process. Naming includes perceptual and conceptual encoding, lemma retrieval (also called lexical selection), word-form encoding, and articulation (e.g., Levelt et al., 1999). Word finding consists of lemma retrieval and word-form encoding. This process normally unfolds rapidly, but in aphasia it is slower and overly susceptible to contextual noise, resulting in increased naming reaction time (RT) or errors. Although several computational models have been developed for word finding (e.g., Dell et al., 2013, Ueno et al., 2011), the WEAVER++ model is the only computational model that explains both RT and accuracy data (e.g., Levelt et al., 1999, Roelofs, 2014).

In everyday life, word finding does not occur in isolation, but usually happens in noisy environments, like while hearing other people speaking or while other semantically related words are activated in the mind of a speaker. The effect of contextual noise on picture naming is typically examined using a picture-word interference (PWI) paradigm (e.g., Glaser & Düngelhoff, 1984). Participants name pictures with written distractor words superimposed or while hearing spoken distractor words. Effects of distractor words have been examined extensively in healthy speakers and also increasingly often in people with aphasia. For example, Piai et al. (2016) used the PWI paradigm to examine word finding in stroke patients and healthy controls, and observed that distractor words caused longer naming RTs and larger interference especially in the patient group. Few studies have used PWI to examine individuals with PPA. Thompson et al. (2012) instructed individuals with lv-PPA and nfv-PPA to name pictures (e.g., of a dog) while trying to ignore superimposed semantically related words (e.g., *mouse*) or unrelated words (e.g., *door*). They observed an abnormally large semantic interference effect on naming RT in these patients. Their results suggest that word finding in nfv- and lv-PPA patients is overly vulnerable to interference from semantic competitors. Vandenberghe et al. (2005) found abnormal interference effects in individuals with PPA, even though no word comprehension deficits were noted on behavioural testing. Similar to Thompson et al., they concluded that selection among competing words belonging to the same semantic category is abnormal in PPA. While these studies suggest that word finding is unusually vulnerable to contextual noise in lv- and nfv-PPA, no study to date has assessed this in all three PPA variants.

In addition to behavioural analysis of the PWI task, the involvement of white-matter (WM) tracts in word finding has recently gained attention. Several studies suggest that a ventral pathway (i.e., fibre tracts running under the Sylvian fissure from posterior occipital and temporal cortex to anterior temporal and frontal areas) is involved in word finding and interference control. In an intraoperative PWI experiment, Ries et al. (2019) observed that stimulation of the ventral pathway induced semantic paraphasias (cf. Sierpowska et al., 2019), causing the participants to name semantically related words instead of the target word. Both lesion and functional magnetic resonance imaging (fMRI) studies contrasting a distractor word condition to a neutral condition (i.e., a row of Xs) have related the increased interference effect to the prefrontal cortex (Piai et al., 2016, de Zubicaray et al., 2001), which is a termination site for the ventral pathway (Catani & Thiebaut de Schotten, 2008). Evidence indicates that ventral tracts play an important role in mediating visual-semantic information processing and its top-down control (e.g., Forkel et al., 2014, Hau et al., 2017, Herbet et al., 2018, Panesar et al., 2018). Yet, the involvement of specific ventral tracts, like the uncinate fasciculus (UF), the inferior fronto-occipital fasciculus (IFOF), and the inferior longitudinal fasciculus (ILF), in naming has remained unclear.

Relevant for picture naming and word reading, the left ILF transmits visual information in occipital cortex to the lexical-semantic language network in temporal cortex, which includes conceptual information in the anterior temporal lobe (e.g., Herbet et al., 2018). The IFOF transmits visual information in occipital cortex to frontal cortex, where it generates awareness and triggers top-down control of visual processing (e.g., Forkel et al., 2014). The UF transmits conceptual information in the anterior temporal lobe to frontal cortex, where it triggers top-down control of semantic processing, referred to as semantic control (e.g., Harvey et al., 2013). Evidence from intraoperative electrical brain stimulation during picture naming suggests that the ILF/UF and IFOF provide (parallel) ventral pathways for relaying visual information in occipital cortex to frontal cortex (Duffau et al., 2009, Mandonnet et al., 2007). Moreover, Herbet et al. (2016) examined naming after surgical removal of low-grade glioma in patients, and observed that damage of the ILF impaired naming performance. However, in examining individuals with PPA, Wilson et al. (2011) did not observe an association between atrophy of the UF and IFOF and naming performance after adjusting for PPA variant and severity of overall cognitive decline. Similarly, Marchina et al. (2011) also did not find a relation between naming performance and the extent of damage to the IFOF and UF in post-stroke aphasia. The difference in results between studies concerning the role of the ventral tracts in naming points to the need for further investigation. Wilson et al. and Marchina et al. only assessed naming accuracy, as is typically done, whereas Herbet et al. examined both naming accuracy and RTs, although they employed a low-precision manual method for the latter. It remains possible that the studies of Wilson et al. and Marchina et al. lacked power to detect an effect. Moreover, it may matter how fibre tract integrity is assessed.

The vast majority of diffusion-weighted imaging (DWI) tractography studies in PPA, like Wilson et al. (2011), has used diffusion tensor imaging (DTI) to measure the structural integrity of white matter, with quantitative comparisons using voxel-averaged metrics, like fractional anisotropy (FA) and mean diffusivity (MD). Since parameters like FA and MD are voxel‐based, and WM voxels are typically occupied by multiple crossing fibres (Jeurissen et al., 2013), these measures are, however, not fibre-specific. To resolve this issue, a recent technique has been introduced, referred to as fixel-based analysis (FBA; Raffelt et al., 2017). FBA enables fibre tract-specific statistical analysis, in which a ‘fixel’ refers to a specific fibre population within a voxel (Raffelt et al., 2015). For each fixel, three FBA metrics can be calculated to assess tissue micro‐ and macrostructure (Raffelt et al., 2017): (1) fibre density (FD) is a microstructural metric that serves as a proxy for density of axons within a fixel; (2) fibre cross-section (FC) is a macrostructural metric that estimates size of the fibre bundle; and (3) fibre density and cross-section (FDC) is a combined measure of FD and FC that represents changes to both micro- and macrostructure. As FDC covers both micro- and macrostructural properties of a tract, it can be regarded as an overall measure of tract integrity.

In the present study, we examined word finding in all three PPA variants and in controls without language impairment using PWI and tractography. We not only assessed naming accuracy, but also RT, which has rarely been done before. Pictures (e.g., of a dog) were named in semantically related word (e.g., *mouse*) and neutral (i.e., a row of Xs) noise conditions. Fixel-based DWI tractography analyses were conducted to assess integrity of the ventral tracts (UF, IFOF, and ILF) in relation to the naming performance. As FDC can be regarded as an overall measure of the tract integrity, we used this as our measure of atrophy. Moreover, we conducted computer simulations with WEAVER++ (e.g., Levelt et al., 1999, Piai et al., 2014, Roelofs, 1992, Roelofs, 2003, Roelofs, 2008, Roelofs, 2018), a computationally implemented psycholinguistic model of the processes underlying word finding, to test our account of the results in terms of reduced propagation of noise when tract integrity is low.

## 2 Materials and methods

### 2.1 Participants

Twenty-three patients diagnosed with PPA and twenty cognitively unimpaired controls were included in this study. The clinical and demographic characteristics of the PPA and cognitively unimpaired control groups are presented in Table 1. Individuals with PPA were recruited from multiple Medical Centers in the Netherlands (Radboud University Medical Center in Nijmegen; Erasmus MC University Medical Center in Rotterdam; Jeroen Bosch Hospital in ‘s-Hertogenbosch; Maastricht University Medical Center in Maastricht). Clinical diagnoses were established based on an extensive multidisciplinary assessment including neuropsychological assessment, neurological testing, and neuroimaging. The individuals with PPA were between 52 and 82 years of age. Specific variants were diagnosed according to the guidelines by Gorno-Tempini et al. (2011). Ten of the patients were classified as lv-PPA, six as nfv-PPA, and seven as sv-PPA. The cognitively unimpaired controls were between 61 and 75 years of age and had neither cognitive impairment (Montreal Cognitive Assessment [MoCA] score ≥ 26) nor self-reported cognitive complaints. There were no significant between-group differences in age (*U* = 194.5, *Z* =  −0.87, *p* = 0.38) and education level (*U* = 155, *Z* =  −1.9, *p* = 0.056). MoCA scores showed significant differences between the groups, which emanated from the cognitively unimpaired controls by definition having significantly higher MoCA scores compared to the PPA group (*U* = 13.5, *Z* =  −5.22, *p* < 0.001, *r* =  −0.81). All participants were right-handed, native Dutch speakers, and gave written informed consent before participation. All patients were tested with the approval of the local ethics committee (CMO Arnhem-Nijmegen, CMO 2016-2340, NL56842.091.16) and healthy controls were tested with the general ethics approval of this committee (“Imaging Human Cognition”, CMO 2014/288).

**Table 1:** Characteristics of included participants.

	PPA				Controls
	All	sv-PPA	lv-PPA	nfv-PPA	
*N*	23	7	10	6	20
Age	69.9 (5.7)	69.6 (4.3)	70.2 (7.7)	69.7 (3.8)	68.4 (5.7)
Number of males/females	14/9	2/5	8/2	4/2	13/7
Education level (1–7)^a^	5.0 (1.0)	5.1 (1.2)	4.8 (0.9)	5.2 (2.0)	5.6 (0.7)
Years of symptoms	3.5 (2.0)	3.6 (1.1)	3.7 (2.9)	2.8 (2.0)	N/A
MoCA score	20.7 (3.6)	21.1 (3.2)	20.3 (4.1)	20.7 (3.9)	27.8 (1.4)
SYDBAT naming score	18.6 (5.5)	12.1 (2.7)	20.8 (2.8)	22.3 (4.9)	27.5 (1.3)

*Note.* Data are reported as mean (*SD*) or number. sv-PPA = semantic variant PPA; lv-PPA = logopenic variant PPA; nfv-PPA = nonfluent variant PPA; *N* = number of participants; MoCA = Montreal Cognitive Assessment; SYDBAT = Sydney Language Battery-NL. ^a^1 = less than six years elementary school; 2 = six years elementary school; 3 = more than six years elementary school; 4 = vocational training; 5 = community college; 6 = advanced vocational training; 7 = Bachelor of Science or higher.

### 2.2 Task and materials

**Fig. 1:**
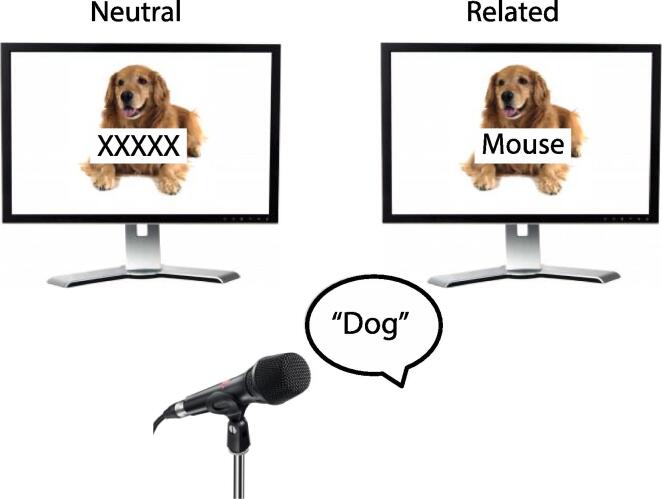
Example of a trial in the two noise conditions of the picture-word interference task.

A picture-word interference task was used to measure word finding. Six animal pictures and six fruit pictures (animals: cow, fish, horse, chicken, dog, mouse; and fruit: strawberry, apple, pear, lemon, banana, kiwi) were used as stimuli. All picture names were Dutch, but the examples in this article are given in English. In the related condition, a different word from the same semantic category as the picture name was superimposed as distractor (e.g., the word *mouse* for the picture of a dog). In the neutral condition, a row of Xs matched for length (number of letters) to the related condition was superimposed on the picture. These conditions correspond to those of the classic colour-word Stroop test, in which participants have to name the ink colour of incongruent colour words (e.g., the word *green* in red ink, say “red”) or the colour of a neutral row of Xs. An example of the stimuli in the two noise conditions of the present study can be found in Fig. 1. There were 60 trials for each condition, with the related condition including each picture with all other picture names of the same category as distractor words. Stimuli were pseudorandomised with the following constraints: A picture stimulus could not be shown twice in a row, a distractor could be shown twice in a row, and a maximum of four items of the same category could be shown in a row. Each participant received a unique randomised order of stimuli.

### 2.3 Procedure

Stimuli were presented in six blocks of twenty trials using Presentation software (http://nbs.neurobs.com). The pictures appeared in the centre of a screen with a white background for 4000 ms. Between trials, a blank screen was presented for 1000 ms. Spoken responses were recorded for later determination of accuracy and RT. First, participants were familiarised with the pictures to be named by showing them the pictures and the appropriate response names. This was done to prevent the RT to depend too much on word finding difficulties and to prevent high patient distress. Then, patients completed twelve practice trials including the same stimuli that were used in the experimental blocks, with every picture presented once. After this, participants could ask clarification questions about the experiment. The instruction to the participants was to name the pictures as quickly and accurately as possible while trying to ignore the superimposed words or Xs.

### 2.4 Statistical analysis of PWI data

Behavioural analyses were performed on both RT and accuracy of the recorded responses. RTs were visually and manually assessed using Praat software (Boersma & Weenink, 2018). Trials that were defined as errors contained hesitations, semantic and phonological paraphasias, naming of the distractor word, or no response. Details on the error classification are shown in supplementary Table 1. Trials in which the naming response included a determiner or trials in which the answer was correct but the reaction time was not reliable were excluded from the RT and error analyses. One participant with lv-PPA was excluded for all further behavioural and tractography analyses because of extremely long RTs (>2 SD above PPA group mean). In total, twenty-two patients diagnosed with PPA and twenty cognitively unimpaired controls were included in the analyses.

Statistical analyses were performed with R version 3.5.0 (R Core Team, 2013), using the lme4 package for mixed-effects models (Bates et al., 2015). Single-trial RTs were analysed with a linear mixed-effects model and errors with a mixed-effects logistic regression (Baayen et al., 2008). For both analyses, the same model structure was used for the fixed effects, in which group (controls, patients) and noise condition (related, neutral) were included as fixed effects, as well as their interaction. A main effect of condition served as a measure of the interference effect. For the overall RT and accuracy models (PPA vs. controls) and the model for RT per subtype, a random effect of condition by participant was included in the analyses. For the analysis of accuracy per subtype, a by-participant random-intercepts only model was used as the more complex model failed to converge. Similar models, with the fixed effect for group replaced by a fixed effect for subtype (control, sv-PPA, lv-PPA, or nfv-PPA) were used to assess RT and error differences between subtypes. While these analyses per subtype were included to present a complete picture, they should be interpreted with caution due to the relatively low number of patients per subtype.

To assess the value of RT measures over and above standard off-line accuracy measures of naming, the relation between PWI RT and scores on both the Boston Naming Test (BNT; Van Loon-Vervoorn and Van der Velden, 2006) and the Sydney Language Battery-NL (SYDBAT-NL) Naming subtest (Eikelboom et al., 2017) was assessed within the PPA group by use of linear mixed-effects models. In these models, noise condition and either the individuals’ BNT or SYDBAT-NL score were included as fixed effects, as well as their interaction, and by-participant random intercepts.

### 2.5 Image acquisition

Structural and diffusion-weighted images were acquired in a single session using a Siemens Prisma Fit 3 T scanner and a 32-channel head coil at the Donders Center for Cognitive Neuroimaging, Nijmegen. Diffusion weighted images were acquired with a simultaneous‐multislice diffusion‐weighted Echo Planar Imaging (EPI) sequence. Acquisition parameters were the following: multiband factor = 3; TR (repetition time) = 2282 ms; TE (echo time) = 71.2 ms; in-plane acceleration factor = 2; voxel size = 2 × 2 × 2 mm^3^; 9 unweighted scans; 100 diffusion-encoding gradient directions in multiple shells; b-values = 1250 and 2500 s/mm^2^; Taq (total acquisition time) = 8 min 29 s. A high-resolution T1 anatomical scan was obtained for spatial processing of the DWI data using the MP2RAGE sequence (Marques et al., 2010) with the following parameters: 176 slices, voxel size = 1 × 1 × 1 mm^3^, TR = 6 s, TE = 2.34 ms, Taq = 7 min 32 s.

### 2.6 Diffusion image processing

DWI images were preprocessed to realign and correct for eddy-current (SPM12) and for artefacts from head and/or cardiac motion using robust tensor modelling (PATCH; Zwiers, 2010). Further analysis steps were performed in MRtrix3 (www.mrtrix.org) and included spatial up-sampling to 1.3 mm^3^ and estimation of the fibre orientation distributions (FOD) at each voxel using multi-tissue constrained spherical deconvolution with a group average response function (Raffelt et al., 2012). After bias field and intensity normalization, a study-specific FOD template was generated and used to register all subjects FOD images to. A combined measure of fibre density and cross-section (FDC) was calculated in template space across all white matter fixels using MRtrix (Raffelt et al., 2017).

### 2.7 Statistical analysis of DWI data

To identify regions with altered FDC in the cognitively unimpaired control and PPA groups and investigate the relationship with PWI interference parameters, fixel-based statistical analysis was carried out using MRtrix. Connectivity-based smoothing, correction for multiple comparisons, and statistical inference was performed using connectivity-based fixel enhancement (CFE) with default smoothing parameters (smoothing = 10 mm full-width at half-maximum, C = 0.5, E = 2, H = 3) and 5000 permutations (Raffelt et al., 2015).

A tract-of-interest analyses was performed to investigate potential degeneration of the ventral fibre pathways, including the ILF, UF, and IFOF. For each tract, appropriate streamlines were selected from the template tractogram based on prior anatomical knowledge, and used to create a fixel‐mask. Mean FDC was calculated for each tract of interest, by averaging over all streamlines within the fixel-masks per tract.

Two tract-of-interest analyses were performed with R version 3.5.0 (R Core Team, 2013). First, between-group differences in FDC of all three tracts were analysed with Kruskal-Wallis tests. Then, to assess the relation between ventral white matter integrity and single-trial RTs, linear mixed-effects models were used for each tract separately, in which group (controls, patients), noise condition (related, neutral), and FDC (continuous) were included as fixed effects, as well as their interaction. Fixel-based FDC measures per tract were incorporated into the mixed-effects models with FDC as an additional fixed effect (continuous) to assess the relation between ventral white matter integrity and RTs in PWI. The models included by-participant random intercepts.

### 2.8 Computer simulations

**Fig. 2:**
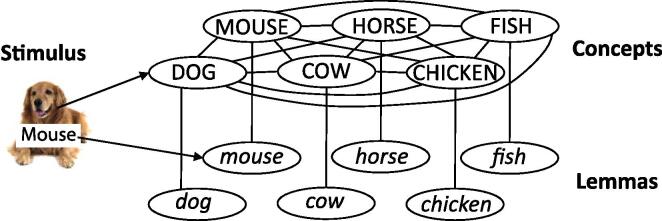
Illustration of the lexical network of the WEAVER++ model.

The simulation protocol and model parameters were exactly the same as in earlier simulations using WEAVER++ (e.g., Levelt et al., 1999, Piai et al., 2014, Roelofs, 1992, Roelofs, 2003, Roelofs, 2008, Roelofs, 2018). The simulations included the same stimuli for a semantic category as in the real experiment (e.g., dog, mouse, fish, horse, cow, and chicken). Each word was represented by a lemma node and a connected concept node, which was connected to all other concept nodes of the same semantic category. The lexical network is illustrated in Fig. 2. In the simulations, a picture activated the corresponding concept node and a printed word activated the corresponding lemma node (related condition). A row of Xs yielded no network activation (neutral condition). Whereas the picture activated the network until the selection of a lemma node, the distractor word activated its lemma node for a limited period of time, the distractor duration parameter. Processing in the model proceeded through time in discrete time steps. On each time step, activation spread through the network following a linear activation function with a decay factor. Activation of nodes in the network triggered the application of condition-action rules. A lemma node was selected as response when its level of activation exceeded that of the other lemma nodes by some critical amount, the selection threshold parameter.

In the simulations, the selection threshold was set to 1.6 for both groups. The distractor duration was set to 75 ms for the control group and to 250 ms for individuals with PPA. With these values, the model captures the empirically observed mean interference effect for each group (real Control = 120 ms, PPA = 277 ms; simulated Control = 114 ms, PPA = 291 ms), indicating overall poorer visual-semantic information processing and top-down control for PPA than for controls. All other parameter values were fixed and identical to those of Roelofs, 1992, Roelofs, 2003. To simulate the effect of ventral tract integrity on lemma retrieval, the strength of the picture and word input to the network was manipulated, which can be taken to capture the functionality of the ventral tracts. Lower strength would correspond to reduced transmission of visual information to the network (representing the role of the ILF) and its top-down control (representing the role of the IFOF and UF). To capture the empirically observed wider range of FDC values for the individuals with PPA than for the controls, strength varied between 1.0 and 0.1 in the simulations of individuals with PPA and between 1.0 and 0.5 for the control group. The mathematically expected lemma retrieval latency in the related and neutral conditions was computed for each strength value for the patients and the controls as specified in all earlier publications on the model.

## 3 Results

### 3.1 Picture-word interference

**Fig. 3:**
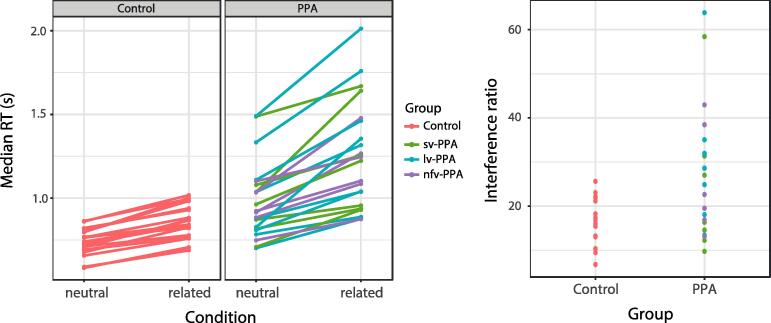
Individual median response time per noise condition for each group (left) and interference effect as percentage increase in RT relative to the neutral condition, i.e., (RT related – RT neutral)/RT neutral (right). RT = response time.

As expected, individuals with PPA were slower than controls (*t* = 5.13, *p* < 0.001, see Fig. 3, left panel) in both noise conditions. An overall interference (related vs. neutral) effect in RT was observed (*t* = 5.00, *p* < 0.001) and the interference effect was statistically larger for the individuals with PPA than for controls (*t* = 3.68, *p* < 0.001, see Fig. 3, right panel). Individual-averaged RTs and interference effects are shown in Fig. 3. Details on the statistics are shown in Table 2.

**Table 2:** Results of the inferential statistics for the response time (top) and accuracy (bottom).

**Measure**	**Statistic**			
**Response time**	**b**	**SE**	***t* (df)**	***p***
PPA vs. controls	0.316	0.0062	5.13 (39)	<0.001
Related vs. neutral	0.137	0.027	5.00 (37)	<0.001
Related vs. neutral: PPA vs. controls	0.141	0.038	3.68 (38)	<0.001
Sv-PPA vs. controls	0.332	0.089	3.74 (37)	<0.001
Lv-PPA vs. controls	0.351	0.081	4.32 (37)	<0.001
Nfv-PPA vs. controls	0.245	0.094	2.60 (37)	0.013
Related vs. neutral: sv-PPA vs. controls ^†^	0.081	0.054	1.50 (37)	0.142
Related vs. neutral: lv-PPA vs. controls ^†^	0.190	0.049	3.85 (37)	<0.001
Related vs. neutral: nfv-PPA vs. controls ^†^	0.136	0.058	2.35 (38)	0.024
**Accuracy**	**b**	**SE**	***z***	***p***

PPA vs. controls	−2.976	0.703	−4.234	0.001
Related vs. neutral	−1.800	0.549	−3.276	<0.001
Related vs. neutral: PPA vs. controls	0.617	0.542	1.138	0.255
Sv-PPA vs. controls	−3.229	0.770	−4.197	<0.001
Lv-PPA vs. controls	−2.513	0.738	−3.406	<0.001
Nfv-PPA vs. controls	−3.256	0.797	−4.086	<0.001
Related vs. neutral: sv-PPA vs. controls ^†^	0.754	0.561	1.344	0.179
Related vs. neutral: lv-PPA vs. controls ^†^	0.319	0.553	0.577	0.564
Related vs. neutral: nfv-PPA vs. controls ^†^	0.797	0.558	1.429	0.153

*Note.* Results obtained from the full model, unless stated otherwise. Results from the group models are indicated by ^†^. SE = standard error.

When the same analysis was performed on PPA subtype level, all subtypes were slower than controls (all *t-*values > 2.6, all *p*-values < 0.05). The interference effect was, however, statistically larger only for lv-PPA (*t* = 3.86, *p* < 0.001) and nfv-PPA (*t* = 2.35, *p* = 0.024) compared to controls. There was no significant difference in interference effect for sv-PPA compared to controls (*t* = 1.51, *p* = 0.14).

The statistical models that assessed the relation between the RT of the PWI task and the accuracy measures of both the BNT and SYDBAT-NL Naming subtest in individuals with PPA showed a main effect of condition (*t* = 3.31, *p* < 0.001; *t* = 3.89, *p* < 0.001, respectively), but not of either BNT or SYDBAT-NL Naming accuracy (*t* = 0.47, *p* = 0.64; *t* = −0.12, *p* = 0.90, respectively). This finding indicates that overall accuracy on standardized naming tests is not a strong predictor of picture naming RT in PWI. A post-hoc linear model was calculated between PWI mean accuracy and PWI mean RT for individuals with PPA, which showed PWI accuracy to predict PWI RT (*R*^2^ = 0.18, *F*(1,20) = 5.66, *p* = 0.028). This relationship likely reflects a severity effect, such that patients that are faster in naming also make fewer errors.

**Fig. 4:**
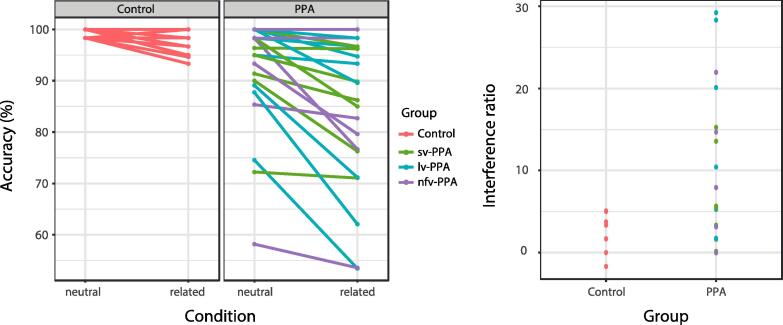
Individual-participant accuracy per noise condition for each group (left) and interference ratio relative to the neutral condition (right). The interference ratio was calculated based on the percentage of correct responses over all valid responses for each noise condition separately. Then, the ratio relative to the neutral condition was calculated as (((accuracy percentage related – accuracy percentage neutral)/accuracy percentage neutral) * −1). The sign of the ratio was inverted for visualization purposes such that positive ratios indicate proportionally more errors in the related than in the neutral condition.

Regarding accuracy, the participants with PPA made more errors compared to controls (*z* = −4.23, *p* < 0.001, see Fig. 4, left panel) and overall errors were more frequent in the related than in the neutral condition (*z* = −3.28, *p* = 0.001). There was no significant difference in interference effect between the PPA and healthy control groups (*z* = 1.14, *p* = 0.255, see Fig. 4, right panel). Individual-averaged accuracy rates and interference effects are shown in Fig. 4. Details on the statistics are shown in Table 2.

On PPA subtype level, all subtypes made more errors than controls (all *z-*values < −3.4, all *p*-values < 0.001) and overall errors were more frequent in the related than neutral condition (*z* = −3.05, *p* = 0.002). There was, however, no significant difference in accuracy-related interference effect between the PPA subtypes and healthy controls (all *z-*values < 1.4, all *p*-values > 0.15).

### 3.2 Tract of interest analysis

#### 3.2.1 FDC comparison between groups

**Fig. 5:**
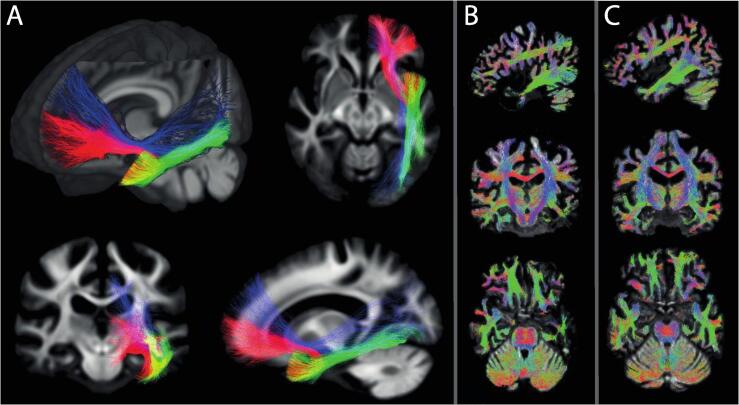
(A) Tract maps of the inferior longitudinal fasciculus (ILF; green), inferior fronto-occipital fasciculus (IFOF; blue), and uncinate fasciculus (UF; red) used to acquire mean fibre density and cross-section (FDC) measures per tract per participant. (B) Whole-brain tractogram of a patient with sv-PPA. (C) Whole-brain tractogram of a cognitively unimpaired control participant. (For interpretation of the references to colour in this figure legend, the reader is referred to the web version of this article.)

**Fig. 6:**
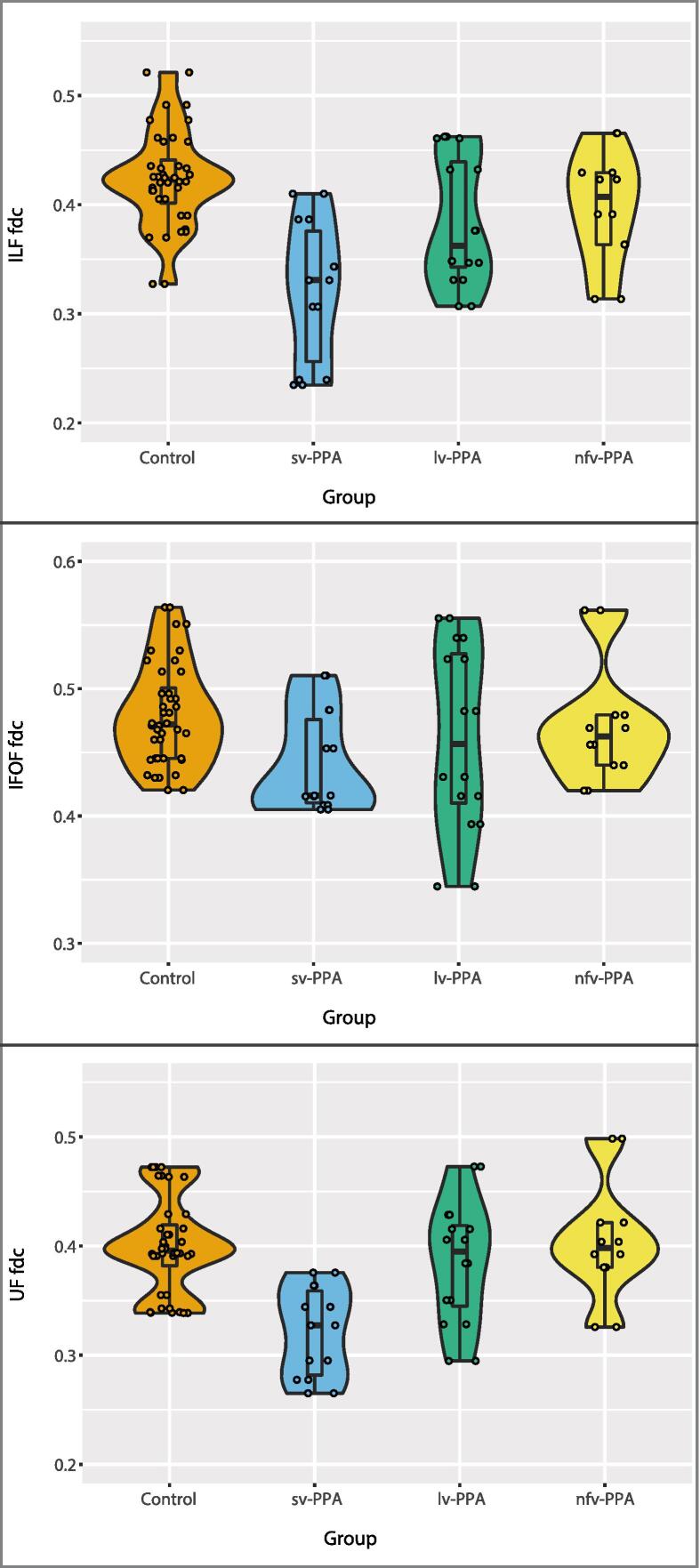
Violin plots of the mean fibre density and cross-section (FDC) for the inferior longitudinal fasciculus (ILF; top), inferior fronto-occipital fasciculus (IFOF; middle), and uncinate fasciculus (UF; bottom) for each PPA variant and the controls. The outer shapes represent the distribution of individual data (indicated by dots), the thick horizontal line inside the box indicates the median, and the bottom and top of the box indicate the first and third quartiles.

The fixel‐masks of the ILF, IFOF, and UF and example tractograms are presented in Fig. 5. Patients with PPA showed a decreased FDC compared to controls for the ILF (χ^2^(1) *=* 6.8027, *p* = 0.009). Between-group FDC differences were not significant for the UF (χ^2^(1) *=* 2.8741, *p* = 0.090) and the IFOF (χ^2^(1) *=* 2.5313, *p* = 0.111). Fig. 6 provides an overview of FDC measures per tract per PPA subtype.

#### 3.2.2 Association of FDC differences with RT of PWI

**Fig. 7:**
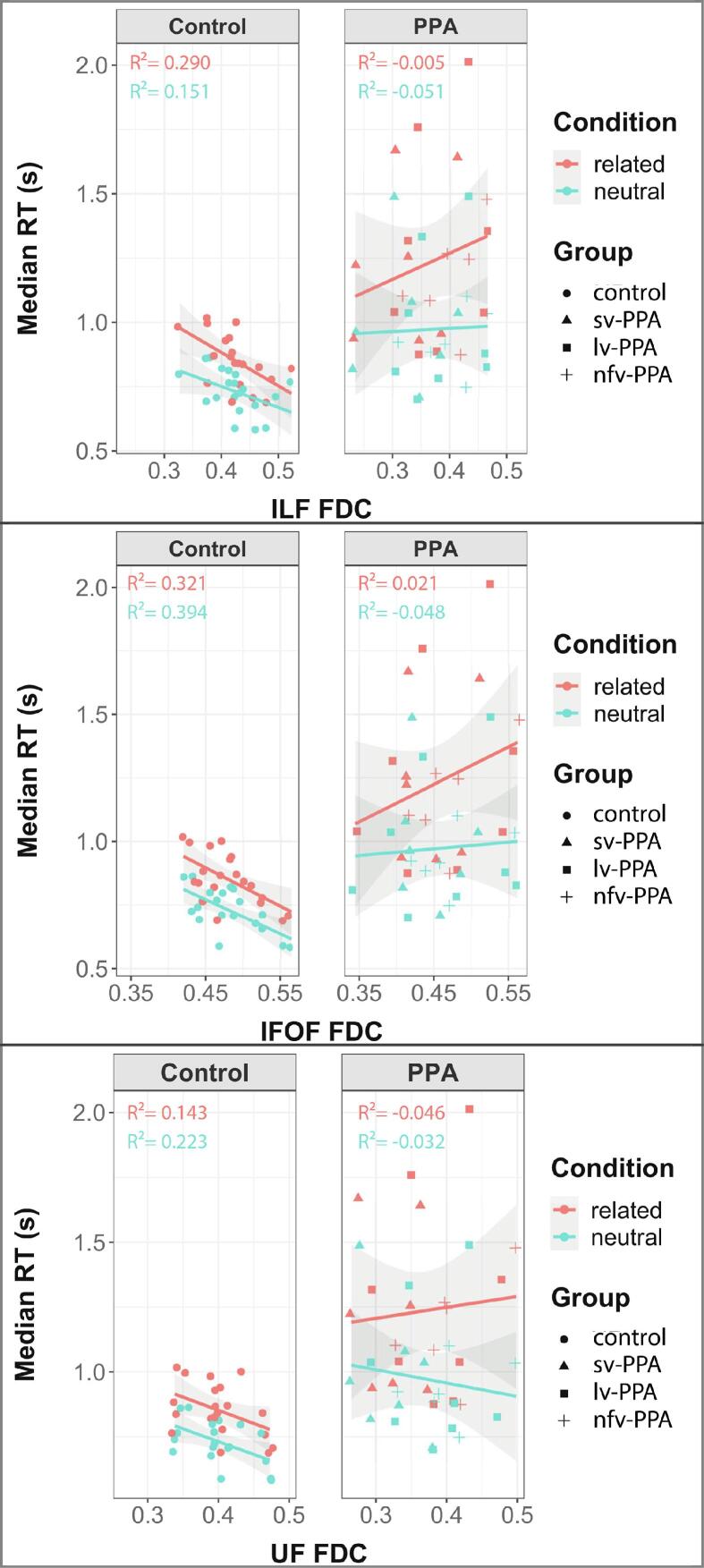
Fibre density and cross-section (FDC) and reaction time (RT; in seconds) per group per condition for the inferior longitudinal fasciculus (ILF; top), inferior fronto-occipital fasciculus (IFOF; middle), and uncinate fasciculus (UF; bottom). Each dot corresponds to a participant. Lines depict the best-fitting linear regression line to the data and shaded areas indicate 95% CI. RT = response time. s = second.

The linear mixed-effects model relating PWI RT to white-matter atrophy showed a three-way interaction between condition, FDC, and group for the ILF (*t* = 3.27, *p* = 0.001), the UF (*t* = 2.44, *p* = 0.015), and the IFOF (*t* = 2.15, *p* = 0.032). This suggests group differences in the effect of condition and FDC on RT and allows for the exploration of this relationship within each group. Fig. 7 provides an overview of the data used in this analysis, presenting FDC and RT per group and condition. Details on the statistics are shown in Table 3.

**Table 3:** Results of the inferential statistics for the response times (RT) analysis per tract.

**RT effect by tract integrity**	**b**	**SE**	***t* (df)**	***p***
**RT effect by ILF integrity**				
FDC * Related vs. neutral: PPA vs. controls	1.082	0.331	3.27 (4433)	0.001
FDC * Related vs. neutral: controls ^†^	−0.327	0.143	−2.286 (2344)	0.022
FDC: neutral in controls ^†^	−0.830	0.412	−2.012 (18)	0.060
FDC: related in controls ^†^	−1.157	0.467	−2.478 (17)	0.023
FDC * Related vs. neutral: PPA ^†^	0.755	0.255	−2.958 (2090)	0.003
FDC: neutral in PPA ^†^	0.395	0.914	0.432 (18)	0.671
FDC: related in PPA ^†^	1.291	1.206	1.070 (18)	0.298
**RT effect by IFOF integrity**				
FDC * Related vs. neutral: PPA vs. controls	0.811	0.377	2.152 (4433)	0.032
FDC * Related vs. neutral: controls ^†^	0.145	0.160	0.909 (2344)	0.363
FDC: controls ^†^	−1.442	0.441	−3.268 (19)	0.004
FDC * Related vs. neutral: PPA ^†^	0.957	0.303	3.153	0.002
FDC: neutral in PPA ^†^	0.616	1.063	0.58	0.569
FDC: related in PPA ^†^	1.702	1.396	1.219 (18)	0.238
**RT effect by UF integrity**				
FDC * Related vs. neutral: PPA vs. controls	0.862	0.354	2.437 (4433)	0.015
FDC * Related vs. neutral: controls ^†^	0.195	0.147	1.330 (2344)	0.184
FDC: controls ^†^	−1.004	0.454	−2.212 (18)	0.040
FDC * Related vs. neutral: PPA ^†^	1.057	0.296	3.574 (2090)	<0.001
FDC: neutral in PPA^†^	−0.494	0.994	−0.497 (19)	0.625
FDC: related in PPA ^†^	0.687	1.343	0.511 (19)	0.615

*Note.* Results obtained from the full model, unless stated otherwise. Results from the group models are indicated by ^†^. Only when a significant interaction effect was found, this was followed up with subsequent analyses per group or condition. SE = standard error.

In healthy control participants, RT was predicted by the integrity of the UF (*t* = −2.21, *p* = 0.040) and IFOF (*t* = −3.27, *p* = 0.004), but no interaction with condition was found for these tracts. For the ILF, however, there was no main effect of tract integrity on RT, but there was a significant interaction with condition (*t* = −2.29, *p* = 0.022). This justified separate linear mixed-effects model analyses within each noise condition for the ILF, which revealed an effect of ILF integrity in the related condition (*t* = −2.48, *p* = 0.023) but less so in the neutral condition (*t* = −2.01, *p* = 0.060).

In PPA patients, there was a significant interaction between condition and FDC for all tracts: the ILF (*t* = 2.96, *p* = 0.003), the UF (*t* = 3.57, *p* < 0.001), and the IFOF (*t* = 3.15, *p* = 0.002). However, separate linear mixed-effects models on each condition revealed no effects of FDC per condition within the PPA group (all *t-*values < 1.2, all *p*-values > 0.05). As Fig. 7 shows, the relation between RT and ILF, UF, and IFOF integrity is such that a larger interference effect is observed when tract integrity is higher.

### 3.3 Computer simulation outcomes

**Fig. 8:**
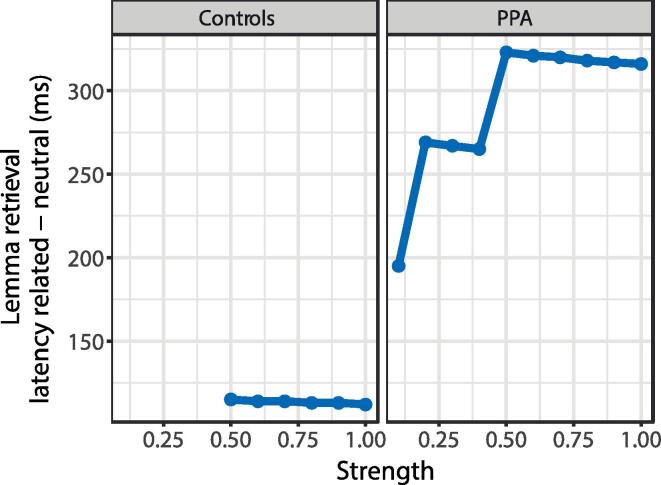
Magnitude of interference on lemma retrieval in WEAVER++ as a function of the duration (control < PPA) and strength of picture and word input. ms = millisecond.

Fig. 8 shows the results of the computer simulations with the WEAVER++ model. The magnitude of the interference effect on lemma retrieval in the model (i.e., related – neutral) is shown as a function of the duration and strength of picture and word input. A longer duration of distractor word input in patients than controls yields larger interference, as empirically observed. Moreover, varying the strength of picture and word input has an effect on lemma retrieval latency depending on the range of variation. In the higher range, reducing strength does not affect the magnitude of interference. However, when strength is further reduced, interference decreases. In the lower range, word noise is propagated less, which reduces interference. A wider range of strength for patients than controls causes interference to decrease with decreasing strength in patients but not in controls, as empirically observed.

## 4 Discussion

In this study, we used PWI to assess word finding and its dependence on ventral tract integrity in people with PPA and cognitively unimpaired controls. We showed that individuals with PPA were overall slower and less accurate in picture naming than controls. Importantly, relative to Xs, semantically related noise words caused more interference in RT for individuals with PPA than for controls. This difference between PPA and controls was reflected in differential relations to tract integrity of the ventral white-matter tracts (i.e., UF, ILF, and IFOF). Whereas the noise effect did not depend much on tract integrity in controls, a lower tract integrity was related to a smaller noise effect in individuals with PPA. In line with this, computer simulations with WEAVER++ showed reduced propagation of noise when input strength is low.

Our results show the value and feasibility of administering a PWI task in individuals with PPA. Most patients were able to perform the task, and RT measures could be obtained. Whereas individuals with PPA were overall less accurate than controls, the effect of related noise on accuracy did not differ between the groups. A difference in interference was only evident when RTs were compared. Thus, the RTs of correct responses have the potential to provide important information that may be missed if only accuracy is examined. The RT measures of the PWI task were unrelated to accuracy scores on standard neuropsychological naming measures like the BNT and SYDBAT-NL Naming subtest. This, again, points to the fact that RT and accuracy assess different and complementary aspects of naming. Thus, RT measures could be a valuable addition in clinical practice uncovering deficits that would otherwise remain undetected (Moritz-Gasser et al., 2012).

No significant relation was observed between the RTs and FDC per condition in PPA, while this was found in controls. This may be explained by the larger variance in the PPA group compared to the controls, as is evident from Fig. 7. This may attenuate correlations in the PPA group and could explain why within the PPA group, the linear mixed-effects analyses revealed an interaction between condition and FDC for all tracts, but (with lower power) no effects per condition.

In line with Thompson et al. (2012), we demonstrated an increased interference in individuals with lv- and nfv-PPA compared to cognitively unimpaired controls. The increased interference in these patients suggests greater overall vulnerability to distractor interference than normal. In addition, PWI was used to test individuals with sv-PPA. Whereas Thompson et al. predicted that patients with sv-PPA should show the same or larger interference due to a degradation of semantic representations, our data seem to suggest the interference found in the present study is smaller for sv-PPA compared to lv- and nfv-PPA.

In addition, we observed that in all PPA variants, lower ventral tract integrity (ILF, UF and IFOF) was associated with a reduced interference effect. This suggests that contextual noise is propagated less when tract integrity is low, and interference will thus be reduced. While it seems paradoxical that decreased structural integrity may have a mitigating effect on noise, a similar finding has been reported by Cope et al. (2017), who found that patients with nfv-PPA perform better than expected on a listening task in a noisy environment and linked this to increased top-down connectivity from frontal to temporal regions during speech perception. In our sample, ventral tract integrity was lowest in sv-PPA, as shown in Fig. 6. Thus, interference is expected to be also smallest in these patients, as our empirical data suggest. The effect of tract integrity on noise propagation may explain why the prediction of Thompson et al. regarding interference in sv-PPA does not seem to hold. To simplify matters in the WEAVER++ simulations, we confined ourselves to an examination of the effect of duration and strength of picture and word input, which concerns visual information processing and its control. However, sv-PPA is known to involve degradation of the anterior temporal lobe and, consequently, a general loss in semantic knowledge (Mummery et al., 2000, Gorno-Tempini et al., 2011). Loss of semantic connections may have further contributed to the reduced effect of the semantically related noise words observed in sv-PPA.

Our tractography approach not only accounts for crossing-fibre populations, but additionally enables a more comprehensive insight into white matter changes by assessing both micro- and macrostructural changes, as illustrated by recent application in other diseases (Raffelt et al., 2017, Vaughan et al., 2017, Mito et al., 2018). Based on micro- and macrostructural properties of each tract, on the group level, our PPA patients showed lower tract integrity only for the ILF compared to controls. Qualitative inspection of integrity per subtype provided a more nuanced picture in which ventral tracts were particularly affected in sv-PPA patients and to a lesser extent in lv-PPA and nfv-PPA patients. This is partly in keeping with previous studies that reported ventral tracts to be mainly affected in sv-PPA (Agosta et al., 2013, Galantucci et al., 2011).

Earlier studies of PPA revealed a spread of diffusion abnormalities beyond sites of local atrophy over time (Agosta et al., 2013, Schwindt et al., 2013). In the current study, patients were included upon diagnosis thereby minimizing the effect of disease progression on spreading of white matter damage, which could explain the absence of evident ventral tract impairments in the lv-PPA and nfv-PPA groups. Still, the heterogeneous symptom duration of our lv-PPA group could explain the variability of ILF integrity in this group (see Fig. 6), as integrity loss of posterior segments of the ILF has been reported in lv-PPA as disease progresses (Tu et al., 2016).

To unravel mechanisms of interference in word retrieval per PPA subtype, information on the time course of the naming process is essential (Thompson et al., 2012). While measures of RT used in this study provide an informative first step in this regard, other time course methods would be necessary to complete the picture. For example, the manipulation of stimulus onset asynchrony, that is, the temporal relation between presentation of the distractor and the picture to be named, can provide additional information about the time windows for specific aspects of speech production (see Thompson et al., 2012). Also, monitoring brain activity with magnetoencephalography (MEG) during PWI could provide information on both location and timing, as MEG yields information on the location of neuronal sources with a temporal resolution in the order of milliseconds (cf. Piai et al., 2014).

While recent studies highlight common neuroanatomical underpinnings related to picture naming in PPA (Leyton et al., 2019, Bruffaerts et al., 2020), the underlying mechanism causing the interference effect might not be the same for the different PPA subtypes (Vandenberghe et al., 2005). The relatively small sample per PPA subtype in the present study limits the statistical power. Therefore, we could not perform all analyses per subtype. Moreover, the subtype-specific findings at the behavioural level warrant replication in a larger scale. However, it should be noted that large study samples of PPA patients are relatively rare given the low prevalence of PPA (Matías-Guiu & García-Ramos, 2013). Still, future studies including larger samples per PPA subtype are required to assess subtype-specific deficits in word finding. This would also allow for the further investigation of the different processes that are suggested to affect naming per subtype, such as phonological encoding and motor preparation of speech in lv- and nfv-PPA (Mack et al., 2013).

In conclusion, our results reveal how word finding depends on ventral tract integrity in individuals with PPA and healthy controls. Although there was no group effect of noise condition on accuracy, compared to rows of Xs, semantically related words increased RT more in patients than controls. This difference in noise effect on RT between groups was differentially related to tract integrity. Whereas the noise effect did not depend much on tract integrity in controls, the effect was smaller when tract integrity was lower in PPA. Computer simulations with WEAVER++ supported an explanation of this paradoxical finding in terms of reduced propagation of noise when tract integrity is low. Our study indicates the significance of the ventral pathway for naming and the importance of RT measurement in PPA.

## Funding

This study was funded by the Gravitation Grant 024.001.006 of the Language in Interaction Consortium from the Netherlands Organization for Scientific Research (NWO) awarded to RPCK and AR. VP is supported by a grant from the Netherlands Organization for Scientific Research (NWO) under award number 451-17-003.

## CRediT authorship contribution statement

**Nikki Janssen:** Conceptualization, Investigation, Methodology, Formal analysis, Writing - original draft, Visualization. **Ardi P.A. Roelofs:** Conceptualization, Funding acquisition, Supervision, Writing - review & editing. **Margot Mangnus:** Conceptualization, Methodology, Writing - review & editing. **Joanna Sierpowska:** Methodology, Writing - review & editing. **Roy P.C. Kessels:** Funding acquisition, Supervision, Writing - review & editing. **Vitória Piai:** Conceptualization, Methodology, Supervision, Writing - review & editing.

## Declaration of Competing Interest

The authors declare that they have no known competing financial interests or personal relationships that could have appeared to influence the work reported in this paper.
