# Supplementary material for: How the speed of word finding depends on ventral tract integrity in primary progressive aphasia
Source: Neuroimage Clin. 2020 Sep 29;28:102450. doi: 10.1016/j.nicl.2020.102450 (PMC7586239; doi:10.1016/j.nicl.2020.102450)
Supplement: Supplementary data 1 [file mmc1.docx]

**Supplementary Table 1.** Error classification

| Error | Description | Example |
| --- | --- | --- |
| Distractor | Response is the distractor word | Target: Chicken  Response: “Dog” (= distractor) |
| Hesitations | Response is preceded by a hesitation marker and/or  initial sounds of the response are slurred/stuttered | Target: Dog  Response: “Euhm..Dog”,  Target: Apple  Response: “Ap..Apple” |
| Semantic paraphasia | Response is semantically related to the target, but is not the distractor. | Target: Strawberry  Response: “Kiwi” |
| Phonological paraphasia | Response contains an unintended phonemic insert, omission, substitution, transposition, or repetition; response shares at least 50% of phonemes with the target | Target: Kiwi  Response: “Si..ki..wi” |
| No response | Time limit was set to 4000ms for response generation | Response: “I don't know”or no response |
| Other | Superordinate: Response reflects category name of target  Circumlocution: Multiword response that describes target | Target: Kiwi  Response: “citrus” (superordinate) |
